# (Phospho)Proteomic dataset of ischemia- and ultrasound- stimulated mouse cardiac endothelial cells in vitro

**DOI:** 10.1016/j.dib.2021.107343

**Published:** 2021-09-04

**Authors:** Uchenna Emechebe, David Giraud, Azzdine Y. Ammi, Kristin L. Scott, Jon M. Jacobs, Jason E. McDermott, Igor V. Dykan, Nabil J. Alkayed, Anthony P. Barnes, Sanjiv Kaul, Catherine M. Davis

**Affiliations:** a Knight Cardiovascular Institute, Oregon Health & Science University, Portland, OR, 97239, USA; b Department of Anesthesiology & Perioperative Medicine, Oregon Health & Science University, Portland, OR, 97239, USA; c Biological Sciences Division, Pacific Northwest National Laboratory, Richland, WA, 99354, USA; d Department of Cell, Developmental and Cancer Biology, Oregon Health & Science University, Portland, OR, 97239, USA

**Keywords:** Ultrasound, Heart, Endothelial cell, Ischemia, Proteomics, Phosphoproteomics

## Abstract

Cardiac endothelial cells respond to both ischemia and therapeutic ultrasound; the proteomic changes underlying these responses are unknown. This data article provides raw and processed data resulting from our global, unbiased phosphoproteomics investigation conducted on primary mouse cardiac endothelial cells exposed to ischemia (2-hour oxygen glucose deprivation) and ultrasound (250 kHz, 1.2 MPa) in vitro [1]. Proteins were extracted from cell lysates and enriched phosphopeptides were analyzed with a high mass accuracy liquid chromatrography (LC) - tandem mass spectrometry (MS/MS) proteomic platform, yielding multiple alterations in both total protein levels and phosphorylation events in response to ischemic injury and ultrasound. This dataset can be used as a reference for future studies on the cardiac endothelial response to ischemia and the mechanistic underpinnings of the cellular response to ultrasound, with the potential to yield clinically relevant therapeutic targets.

## Specifications Table

**Table:** 

Subject	Omics: Proteomics
Specific subject area	Ischemia, therapeutic ultrasound, vascular biology
Type of data	Raw data, MassIVE and ProteomeXchange Supplementary Tables (Excel) of processed data.
How data were acquired	LC-MS/MS analysis on Q Exactive HF Hybrid Quadrupole-Orbitrap mass spectrometer (Thermo Scientific). Identification and quantification of peptide peaks performed using Accurate Mass and Time (AMT) tag approach; LC-MS/MS data was searched using MS-GF v6432 (09/08/2011).
Data format	Raw Analyzed
Parameters for data collection	Proteins were extracted from primary mouse cardiac endothelial cultures either at basal conditions or following stimulation by oxygen glucose deprivation and/ or ultrasound.
Description of data collection	Three independently isolated and cultured primary mouse endothelial cell preparations were each exposed to ultrasound, in vitro ischemia (oxygen glucose deprivation), a combination of the two, or were controls, resulting in 12 samples overall. Cells were harvested and lysed. Proteins were extracted and phosphopeptides enriched then subjected to LC-MS/MS.
Data source location	Institution: Pacific Northwest National Laboratory City/Town/Region: Richland, WA 99354 Country: USA
Data accessibility	All raw data are deposited in MassIVE https://massive.ucsd.edu/ProteoSAFe/dataset.jsp?task=c6e7d47418cc432484f85e14d305c5cb and ProteomeXchange available at http://proteomecentral.proteomexchange.org/cgi/GetDataset?ID=PXD023331
Related research article	Co-submission: U. Emechebe, D. Giraud, A. Y. Ammi, K. L. Scott, J. M. Jacobs, J. E. McDermott, I. V. Dykan, N. J. Alkayed, A. P. Barnes, S. Kaul, C. M. Davis. Phosphoproteomic Response of Cardiac Endothelial Cells to Ischemia and Ultrasound. Biochim Biophys Acta Proteins Proteom. 2021; 1869:140683 [1]

## Value of the Data

• These data represent valuable information on the acute proteomic response of cardiac endothelial cells to ischemia and/ or ultrasound stimulation.
• These data can benefit researchers working on ischemic coronary disease, endothelial contributions to ischemic injury or on mechanisms underlying the effects of therapeutic ultrasound on the heart and vasculature.
• These data may reveal therapeutic targets in ischemic heart disease and offer mechanistic insights into beneficial or off-target effects of therapeutic ultrasound.

## 1 Data Description

This dataset consists of raw proteomic and phosphoproteomic data from 3 primary mouse cardiac endothelial cell (EC) cultures harvested under the following conditions: 1. Control (baseline; BL); 2. In vitro ischemia (2 hr oxygen glucose deprivation; OGD); 3. Ultrasound stimulation (250 kHz, 1.2 MPa; US); 4. OGD + US, generating 12 samples overall. The raw data are deposited in MassIVE (available at https://massive.ucsd.edu/ProteoSAFe/static/massive.jsp), accession number MSV000086652, and ProteomeXchange (available at http://www.proteomexchange.org), accession number PXD023331.

We also provide supplemental processed data listing proteins and phosphopeptides that are significantly altered by OGD and/ or US. Supplementary Table 1 lists all proteins significantly altered when combined across all conditions. Abbreviated protein names for proteins significantly altered within each comparison (i.e. BL vs OGD), and also those shared between 2 comparisons (i.e. BL vs OGD and OGD vs OGD+US) are provide in Supplementary Table 2. Supplementary Tables 3-5 list proteins significantly altered between BL and OGD (Supplementary Table 3), BL and US (Supplementary Table 4) and OGD and OGD+US (Supplementary Table 5) with log2 relative protein abundance, fold change and calculated p values.

Supplementary Table 6 lists all significantly altered phosphopeptides across all groups, with log2 relative phosphopeptide abundance. Significantly altered phosphopeptides are separated out by group comparisons in Supplementary Table 7, i.e those regulated by US or OGD as well as those regulated by both stimuli. Further peptide information and log2 relative abundance values between comparisons (BL vs OGD, BL vs US, OGD vs OGD+US) are provided in Supplementary Tables 8-10, with calculated p values and fold change for each comparison.

## 2 Experimental Design, Materials and Methods

### 2.1 Endothelial culture and sample generation

#### 2.1.1 Endothelial culture reagents

**Table:** 

Product name	Final concentration	Vendor	Catalogue number
**Growth medium**			
DMEM		Thermo Fisher Scientific, Waltham, MA	11965-084
+ glucose	4.5 g/L		
+ L-glutamine	4 mM		
FBS	20 %	Sigma-Aldrich, St. Louis, MO	12103C
ECGS, BT-203	100 µg/mL	Biomedical Technologies Inc., Stoughton, MA	J64516
Gentamicin	50 µg/mL	Sigma-Aldrich	G1397
Heparin	100 µg/mL	Sigma-Aldrich	H3149-100KU
**Other reagents**			
Collagenase CLS2	1mg/mL	Worthington Biological, Lakewood, NJ	LS004176
Deoxyribonuclease I	10 µg/mL		D4513-1VL
D-PBS		Thermo Fisher Scientific	14190-250
Rat anti-mouse CD31 (PECAM-1, clone MEC 13.3) antibody		BD Pharmingen, BD Biosciences, Franklin Lakes, NJ	553369
Rat anti-mouse CD102 (ICAM-2) antibody		BD Pharmingen	553326
Dynabeads, sheep anti-rat IgG		Thermo Fisher Scientific	110-35
Albumin, from bovine serum (fraction V)		Sigma-Aldrich	A4503
Collagen type IV		Sigma-Aldrich	C5533
Trypsin-EDTA, 0.25%		Thermo Fisher Scientific	25200-072
DMEM, no glucose		Thermo Fisher Scientific	11966-025
MicroAmp optical adhesive film		Thermo Fisher Scientific	4311971
cOmplete mini EDTA-free protease inhibitor cocktail	1 tablet/ 10mL	Sigma-Aldrich	11836170001
Phosphatase inhibitor cocktail 2	10 µL/mL	Sigma-Aldrich	P5726
Phosphatase inhibitor cocktail 3	10 µL/mL	Sigma-Aldrich	P0044

#### 2.1.2 Dynabead preparation

Dynabeads were placed on a magnetic separator (DynaMag-2; Life Technologies), buffer aspirated and beads resuspended in PBS supplemented with 0.1% BSA; this process was repeated 3 times to wash the beads. Beads were subsequently suspended in PBS supplemented with 0.1% BSA and incubated with antibody (anti-CD31 or anti-CD102) overnight at 4 °C on a rotator. The next day, the beads were again washed 3 times in PBS + 0.1% BSA, to remove unbound antibody. Antibody-bound beads were finally resuspended in PBS + 0.1% BSA, to a concentration of 4 × 10^8^ beads/ mL, and stored at 4 °C.

#### 2.1.3 Primary mouse heart endothelial cell (EC) culture

Each culture consisted of independent cell isolations using a pool of five 8-week-old male hearts from C57BL6 mice (Charles River Laboratory, Wilmington, MA). Each heart was rapidly harvested, minced with a razor blade and collagenase digested for 45 minutes at 37 °C. During digestion, a 6”-long 14-gauge metal cannula was used to triturate the cells at 15 minute intervals. Fetal bovine serum (10%) supplemented high glucose DMEM was added to terminate digestion. Cells were recovered from the solution by centrifugation and resuspended in DPBS containing rat anti-CD31 antibody bound to sheep anti-rat Dynabeads. This cell solution was rotated for 40 minutes at room temperature which was followed by magnetic selection with Dynabead-bound cells harvested and plated onto T75 flasks pre-coated with collagen and cultured using endothelial growth medium. As cultures reached confluence, they were enzymatically dissociated and resorted using rat anti-CD102 antibody bound sheep anti-rat Dynabeads in the same manner as the first cell sort. CD102 selected cells were grown on collagen-coated T75 flasks; upon confluence, cells were enzymatically detached and passed through the magnetic sorter, cells with bound beads were discarded, the remaining ECs were plated for experiment.

#### 2.1.4 In vitro ischemia: oxygen - glucose deprivation (OGD)

OGD was initiated by switching the ECs from endothelial growth medium to glucose-free DMEM with three DPBS rinses to remove residual serum and glucose. The 12-well cell culture plates containing the ECs were then placed in the anaerobic chamber filled with an anoxic gas mixture consisting of 90% N2, 5% CO2 and 5% H2. ECs were placed in an 37 °C anaerobic chamber (Coy Laboratory Products, Grass Lake, MI) for 120 minutes. A palladium catalyst kept O_2_ at 0 ppm and was monitored throughout using an Oxygen-Hydrogen Gas Analyzer (Coy Laboratory Products). Termination of OGD was achieved by removing cells from the chamber and replacing glucose-free medium with degassed high glucose DMEM, again rinsing cells (x3) with DPBS in between; cells were now ready for ultrasound stimulation.

#### 2.1.5 Ultrasound exposure and sample collection

The 12-well plates containing ECs were filled completely with high glucose DMEM (degassed) and sealed with MicroAmp optical adhesive film, eliminating any air pockets between medium and seal. The plates were placed into an acrylic tank filled with water (37.4 °C, 0.2 µm filtered and degassed). Cellular insonification was accomplished using a single-element high intensity focused ultrasound transducer that was operated by a power amplifier (either 600A225 Amplifier, Amplifier Research, Souderton, PA or RAM 5000, Ritec Inc., Warwick, RI) with an electrical impedance matching network, at 250 kHz (H-171, Sonic Concepts, Bothell, WA). Our transducer was calibrated for 1.2 MPa peak rarefactional acoustic pressure amplitude (PRAPA) at the focus and fixed to a three-axis translation system (Velmex Inc., Bloomfield, NY). The transducer was positioned such that the focus was at the EC surface and during US application it was scanned over the central area (1 cm^2^) of each well, with each scan consisting of 10 back- and-forth 1-cm lines separated by 1 mm with each full well scan lasting approximately 2 minutes. Each well underwent two sequential scans, for each scan the transducer was pulsed with 50 cycles at a 50 Hz (pulse repetition frequency PRF; 1% duty cycle). After US scanning was complete, each plate was removed from the water chamber and held at room temperature until harvest. Cells were harvested 15 after US stimulation as calculated from the time the middle well of a given plate was scanned. Cells not undergoing ultrasound stimulation (i.e. control plates) were treated in the same manner as experimental plates. Briefly, US control plates were also filled with degassed DMEM and placed into the water chamber for the same duration as US-exposed plates. They were then removed from the tank and held at room temperature until harvest, identical to the experimental, US-treated cells. Cells from 24 individual wells were harvested and pooled to generate each sample. Cells were harvested by addition of lysis buffer and cell removal via scraping on ice; lysates were immediately frozen using dry ice and stored at -80 °C. Lysis buffer consisted of 0.1 mM Ethylenediaminetetraacetic Acid (EDTA), 10 mM HEPES, pH 7.9, 10 mM Potassium Chloride, 0.1mM Ethylene Glycol Tetraacetic Acid (EGTA), cOmplete Mini EDTA-free Protease Inhibitor Cocktail, Sigma Phosphatase Inhibitor Cocktail 2, Sigma Phosphatase Inhibitor Cocktail 3, 0.5mM Phenylmethanesulfonyl Fluoride (PMSF) and 0.5% NP40.

### 2.2 Proteomic analysis of EC lysates

#### 2.2.1 Proteomics reagents

Unless otherwise stated, all reagents and chemicals used were obtained from Sigma-Aldrich (St. Louis, MO). Sequencing Grade Modified Trypsin was purchased from Promega (Madison, WI), acetonitrile and ammonium bicarbonate and were sourced from Fisher Scientific (Pittsburgh, PA), and. Pierce Bicinchoninic acid (BCA) assay reagents and protein assay standards were purchased from ThermoFisher Scientific (Waltham, MA); Ni-NTA-agarose beads were purchased from QIAGEN (Valencia, CA); and purified and >18 MW deionized water (Nanopure Infinity ultrapure water system, Barnstead, Dubuque, IA) was used for all solutions and aqueous buffers.

#### 2.2.2 Sample preparation

Cell lysates were thawed and centrifuged at 1000 x g for 30 min, and washed x3 with NH4HCO3, pH 7.8. Following the final spin, cell pellets were homogenized for 5 min using a Qiagen TissueRuptor in buffer of 8 M Urea in NH4HCO3, pH 7.8, to denature the samples. Protein concentration was subsequently determined by Pierce BCA assay (ThermoFisher Scientific).

#### 2.2.3 Protein extraction and trypsin digest

Proteins were reduced and denatured in 8 M Urea in NH4HCO3, pH 7.8 buffer with 5 mM Dithiothreitol (DTT) prior to alkylation of free sulfhydryl groups using 10 mM Iodoacetamide in the dark; each reaction was performed for at 37 °C with constant shaking at 1000 rpm on a Thermomixer R (Eppendorf, Westbury, NY) for 1 hour. Subsequently 50 mM Ammonium Bicarbonate buffer pH 7.8 containing 1 mM CaCl2 was used to dilute samples 8-fold. Tryptic digestion was performed using Sequencing Grade Modified Trypsin, which was prepared according to the manufacturer's instructions. The trypsin was added to the samples at a 50:1 (wt/wt) protein-to-trypsin ratio and samples were incubated at 37 °C for 3 hours in a Thermomixer R. The trypsin digestion was stopped by acidifying samples to 0.1% Trifluoroacetic Acid (TFA) with 10% TFA stock solution following the 3-hour incubation. Samples were centrifuged at 4000 x g for 15 minutes, transferred to a new tube and stored at -80 °C until the next step.

Tryptic peptides were desalted using reversed-phase SCX Solid-Phase Extraction (SPE) columns (Discovery-SCX, SUPELCO, Bellefonte, PA) with 80:20 ACN: 500mM Triethylammonium Bicarbonate (TEAB) for peptide elution. Samples were then acidified to 0.1% TFA, following peptide concentration using a SpeedVac vacuum concentrator (Thermo Savant, Holbrook, NY), the volume of each sample was adjusted to 5 mL. Subsequently, sequential desalting was carried out using appropriate C18 Strata C18-E SPE columns (Phenomenex, Torrance, CA). MeCN: water in a 80:20 ratio was used to elute peptides from the SPE column; peptides were then concentrated in a SpeedVac vacuum concentrator.

Relative peptide concentration was determined by BCA Protein Assay; a 100 ug cleaned peptide aliquot of each sample was dried using a SpeedVac and stored at -80 °C until the next step.

#### 2.2.4 Phosphopeptide enrichment using IMAC

Ni-NTA-agarose beads (QIAGEN, Hilden, Germany) were used to make Magnetic Fe3+-NTA-agarose beads, which were freshly prepared, as previously described [2], [3]. Dry peptides were reconstituted in 80% Acetonitrile, 0.1% TFA IMAC wash/binding buffer and then incubated for 30 minutes with the 5% immobilized bead suspension at room temperature. Next, the beads were washed with the IMAC wash buffer x4. Subsequently, the captured phosphopeptides were eluted from the beads in Acetonitrile: 5% Ammonia in a 1:1 ratio, pH 8.0. The samples were directly eluted into LCMS vials, dried using a SpeedVac and reconstituted in 0.1% TEA, 2% ACN for subsequent LC-MS/MS analysis.

#### 2.2.5 Instrument analysis

All samples, global and phosphopeptide enriched, were subjected to a custom high mass accuracy LC-MS/MS system where the LC component consists of automated reversed-phase columns prepared in-house by slurry packing 3-µm Jupiter C18 (Phenomenex) into 35-cm x 360 µm o.d. x 75 µm i.d fused silica (Polymicro Technologies Inc.), as described previously [4]. The MS component consisted of a Q Exactive HF Hybrid Quadrupole-Orbitrap mass spectrometer (Thermo Scientific) outfitted with a custom electrospray ionization interface. Electrospray emitters were custom made using 360 µm o.d. x 20 µm i.d. chemically etched fused silica capillary. Analysis of the global and phosphoproteome samples applied similar conditions, except that the spray voltage was 2.2 kV for the phosphoproteome samples. All other instrument conditions were set as described previously [4].

### 2.3 Data analysis

The Accurate Mass and Time (AMT) tag approach was used to identify and quantify the peptide peaks detected [5]. Briefly, the LC-MS/MS data were searched using MS-GF v6432 (09/08/2011); peptide search using MS-GF plus was carried out with full tryptic digestion allowing a maximum of two missed cleavages against a mouse UniProt database (version Oct, 5 2016, 26460 entries) for both global and phosphoproteome datasets. For the phosphoproteome results, the acetylation (protein N-term), phospho (STY) and oxidation (M) and were set as variable modifications and the carbamidomethyl (C) was set as a fixed modification. The minimal peptide length for identification was set as 5. A score greater than or equal to 1E-10 was used to filter the results, resulting in a false discovery rate of ∼1% at the level of peptides, this was used to populate the respective AMT tag databases [6], [7]. The LC-MS data was processed using multiple in-house developed informatics tools (publicly available ncrr.pnnl.gov/software) and the resulting LC-MS features were correlated to an AMT tag database containing accurate mass and LC separation elution time information for peptide tags generated from serum proteins. The tools used to achieve this were algorithms for peak-picking and determining charge states and isotopic distributions [8]. Further downstream data analysis incorporated all possible detected peptides into VIPER, a visualization program, to correlate LC-MS features to the peptide identifications in the AMT tag database [9]. Peptide quantification was based on the detected and matched peak intensity profile over elution time as determined in VIPER. Protein level quantification was based on the R-rollup approach, as performed and described in Dante [10](http://omics.pnl.gov). Briefly, R-rollup is a peptide to protein quantitative scaling approach that utilizes a reference peptide for each protein as a scaling factor followed by the median of all scaled peptide abundances towards calculation of the particular protein abundance.

#### 2.3.1 Statistical analysis

Generated relative peptide abundance values were Log2 transformed and subjected to ANOVA analysis and, in Dante, underwent correlation plotting [10](http://omics.pnl.gov). Median peptide intensity values were used to estimate relative protein abundance [11], requiring a minimum of 2 unique peptides. For global analysis, peptide level results were compiled into protein values using the R-rollup approach feature in Dante [10] and then underwent ANOVA analysis and correlation plotting. Statistical comparisons were based upon pval <0.05 criteria across three independent biological samples per condition.

## Ethics Statement

All experiments comply with ARRIVE 2.0 guidelines and were performed according to the National Institutes of Health Guidelines for the Use and Care of Laboratory Animals (NIH Publications No. 8023, revised 1978). Protocols were approved by the Institutional Animal Care and Use Committee of Oregon Health and Science University.

## CRediT authorship contribution statement

**Uchenna Emechebe:** Formal analysis, Investigation, Writing – review & editing. **David Giraud:** Investigation, Writing – review & editing. **Azzdine Y. Ammi:** Investigation, Writing – review & editing. **Kristin L. Scott:** Investigation, Writing – review & editing. **Jon M. Jacobs:** Formal analysis, Investigation, Writing – review & editing. **Jason E. McDermott:** Formal analysis, Investigation, Data curation, Writing – review & editing. **Igor V. Dykan:** Investigation, Writing – review & editing. **Nabil J. Alkayed:** Conceptualization, Writing – review & editing. **Anthony P. Barnes:** Conceptualization, Formal analysis, Writing – review & editing, Supervision, Project administration. **Sanjiv Kaul:** Conceptualization, Methodology, Writing – review & editing, Supervision, Project administration. **Catherine M. Davis:** Conceptualization, Methodology, Formal analysis, Investigation, Writing – original draft, Writing – review & editing, Supervision, Project administration.

## Declaration of Competing Interest

The authors declare that they have no known competing financial interests or personal relationships which have or could be perceived to have influenced the work reported in this article.

## Appendix Supplementary materials

Image, application 1

Image, application 2

Image, application 3

Image, application 4

Image, application 5

Image, application 6

Image, application 7

Image, application 8

Image, application 9

Image, application 10
